# The Effects of Nanoclay on the Mechanical Properties, Carvacrol Release and Degradation of a PLA/PBAT Blend

**DOI:** 10.3390/ma13040983

**Published:** 2020-02-22

**Authors:** Roberto Scaffaro, Andrea Maio, Emmanuel Fortunato Gulino, Marco Morreale, Francesco Paolo La Mantia

**Affiliations:** 1 Department of Engineering, University of Palermo, Viale delle Scienze Ed. 6, 90128 Palermo, Italy; emmanuelfortunato.gulino@unipa.it (E.F.G.); francescopaolo.lamantia@unipa.it (F.P.L.M.); 2 Faculty of Engineering and Architecture, Kore University of Enna, Cittadella Universitaria, 94100 Enna, Italy

**Keywords:** PLA, PBAT, montmorillonite, essential oil, film blowing, green composites, drug release, hydrolytic degradation, mechanical properties, biodegradable polymer blends

## Abstract

The formulation of polymeric films endowed with the abilities of controlled release of antimicrobials and biodegradability is the latest trend of food packaging. Biodegradable polymer (Bio-Flex^®^)-based nanocomposites containing carvacrol as an antimicrobial agent, and a nanoclay as a filler, were processed into blown films. The presence of such hybrid loading, while not affecting the overall filmability of the neat matrix, led to enhanced mechanical properties, with relative increments up to +70% and +200% in terms of elastic modulus and elongation at break. FTIR/ATR analysis and release tests pointed out that the presence of nanoclay allowed higher carvacrol loading efficiency, reasonably hindering its volatilization during processing. Furthermore, it also mitigated the burst delivery, thereby enabling a more controlled release of the antimicrobial agent. The results of mass loss tests indicated that all the formulations showed a rather fast degradation with mass losses ranging from 37.5% to 57.5% after 876 h. The presence of clay and carvacrol accelerated the mass loss rate of Bio-Flex^®^, especially when added simultaneously, thus indicating an increased biodegradability. Such ternary systems could be, therefore, particularly suitable as green materials for food packaging applications, and for antimicrobial wrapping applications.

## 1. Introduction

The rising interest toward eco-sustainable active packaging has been triggered by recently growing environmental concerns [1,2,3]. In fact, issues related to the disposal and non-biodegradability of fossil-derived plastics caused serious water and land pollution, with about 4.8–12.7 million tons of plastics disposed in ocean in the year 2010 [4]. In 2015, more than 6000 million metric tons (Mt) of plastic waste was generated globally, with only about 9% led to recycling and 12% to incineration, while almost 80% was disposed in the ecosystem [5,6].

From that perspective, the choices of materials and processing techniques must satisfy the current demands in terms of environmental sustainability. Using bio-based, biodegradable, and compostable resins may bring several advantages in terms of plastic waste reduction, renewability, and eco-friendliness. Moreover, there is much space available for an increased use of biodegradable/bio-based polymers: for instance, it is reported that only approximately 1% of total global plastic production’s capacities in 2015 were attributable to bio-based and biodegradable plastics [5]. 

Among bioplastics, poly(lactic acid) (PLA) and poly(butylene adipate-co-terephthalate) (PBAT) are the most widespread for food packaging applications. In this context, Bioflex^®^ (BF), a commercial blend of PLA and PBAT [7], is particularly promising as a bio-based polymer material for food packaging applications [8,9,10]. In fact, it is versatile, compostable, recyclable, and moreover, possesses good processability and intriguing mechanical properties, resulting from the combination of the mechanical robustness of PLA and the toughness of PBAT [7,8,9,11,12]. 

Furthermore, the development of materials for food packaging relies on the possibility of releasing antimicrobial additives, able to preserve the organoleptic characteristics of food, while avoiding the growth of bacterial strains and fungi [13]. Aiming to maintain the aforementioned “green” prerequisites, the choice of antimicrobial agents must be oriented toward natural compounds. Indeed, the recent advances and future perspectives in the design of materials for food packaging are oriented towards the tuning and optimization of a combined strategy, involving the use of bioactive additives, biodegradable and/or bionanocomposite matrices, and green technologies [4].

In this context, essential oils have recently gained enormous attention as bioactive additives, owing to their extreme safety and lower environmental effects when compared to metal or metal oxide nanoparticles, such as zinc, silver, copper, and iron oxides, typically used as antimicrobial additives for polymeric films [14,15].

Among natural compounds, carvacrol (2-methyl-5-(1-methylethyl)-phenol), a terpernoid phenol extracted from oregano essential oil, is probably one of the most widespread monoterpenoids, owing to its several biological and pharmacological properties, including anti-inflammatory, antimicrobial, antioxidant, antitumor, and insecticidal activities [13,15].

Of course, the choice of film blowing as a processing technique represents a key-strategy to avoid time-consuming protocols and toxic solvents, so as to be industrially scalable yet green. By contrast, although being currently widespread for industrial production of packaging films of many bioplastics, this technique might display a low degree of carvacrol loading efficiency, due to the high volatility of this latter compound [16,17,18]. Aiming to mitigate the burst release and volatilization of carvacrol from bioplastic during processing, the use of complexing agents was proposed, e.g., β-cyclodextrin, which results in the introduction of an additional step in the fabrication protocols, with obvious repercussions in terms of manufacturing costs and time [19]. Another possible strategy involves nanofillers that display good affinity to both carvacrol and polymer matrix. In this context, graphene and lignin were recently proposed for PLA and PBAT, respectively. However, the safety of the former is still far from being fully elucidated, whereas the latter nanofiller, while being suitable for PBAT and polyphenols, showed scarce affinity to PLA and decomposition temperatures inadequate to be melt-processed with PLA/PBAT blends [20].

Using a natural, nanostructured filler, such as a clay, could represent a successful strategy for inhibiting carvacrol volatilization during processing, and could even modulate its release kinetics and improve the mechanical properties of such systems [21]. 

Herein, we report on the preparation and characterization of ternary systems based on Bioflex^®^, carvacrol, and a nanoclay, namely, Dellite 72T, with a particular focus on some target properties, such as mechanical performance, additive release activity, and degradability.

## 2. Materials and Methods 

### 2.1. Materials

Bioflex (BF), trade name Bio-Flex^®^ F2110, was purchased from FKuR (Willich, Germany). It is a sample of biodegradable polymer blend, whose formulation is proprietary, which is known to contain PLA and a biodegradable co-polyester, and some additives [7,22]. Its main physical properties are: density = 1.27 g/cm^3^, melting temperature = 145 °C, melt flow index = 3–5 (at T = 190 °C and 2.16 kg).

Carvacrol (2-methyl-5-(1-methylethyl)-phenol) was supplied by Sigma Aldrich (St. Louis, MO, USA). It has the following main characteristics: density= 0.9772 g/cm^3^, boiling temperature = 237.7 °C, purity ≥ 97%. 

The nanoclay used was a montmorillonite, modified by dimethyl-dihydrogenated tallow ammonium, Dellite 72T (D72T), supplied by Laviosa Chimica Mineraria (Livorno, Italy).

### 2.2. Preparation

The materials were prepared via film blowing by using a single-screw extruder, Brabender Plasticorder (Duisburg, Germany), having D = 19 mm and L/D = 25, equipped with a film blowing and take-off unit. Details of preparation, including formulations, operating parameters adopted, blow-up ratios (BUR), and final thicknesses of the films are listed in Table 1.

Prior to processing, BF and D72T were vacuum-dried in an oven at 90 °C overnight. Carvacrol loading was accomplished by impregnating dried polymer pellets (for BF-C) or nanoclay (for BF-D-C) with essential oil in a capped flask under vigorous mechanical stirring for at least 2 h prior to feeding to the extruder.

### 2.3. Characterization Techniques

Morphological analysis was carried out by scanning electron microscopy (SEM) technique, using a FEI (Thermo Fisher Scientific, Waltham, MA, USA) QUANTA 200 ESEM on cryofractured specimens. Water contact angle (WCA) measurements were performed at room temperature by means of a FTA 1000 (First Ten Ångstroms, Cambridge, UK) instrument. More in detail, 4 μL of deionized water was dropped onto the surface of each sample by means of an automatic liquid drop dosing system. Images of the drops on the surfaces were acquired after 20 s. 

Mechanical properties of the samples were evaluated through tensile tests, performed using an Instron 3365 (Instron, Norwood, MA, USA) universal testing machine, with a first crosshead speed of 1 mm/min (until 3 min), and then a second crosshead speed increased to 100 mm/min, up to specimen failure (ISO 527-3). Elastic modulus (E) was thus calculated as the initial slope of stress-strain curves; tensile strength (TS) and the elongation at break (EB) were evaluated as the maximum values of stress and strain for each curve. At least 10 specimens were tested for each run.

The structural and chemical composition of each sample was analyzed by Fourier-transform infrared (FTIR) spectroscopy in attenuated total reflection (ATR) mode, using a FT-IR/NIR Spectrum 400 spectrophotometer (Perkin-Elmer, Waltham, MA, USA). Spectra were obtained by collecting 32 scans, in the 4000–400 cm^−1^ wavenumber range (testing at least five different positions for each sample). Furthermore, since carvacrol is characterized by an absorption band at about 812–814 cm^−1^, a focus on the 900–700 cm^−1^ range was conducted to qualitatively assess the content of carvacrol per film unit [23].

Carvacrol release was assessed via UV-vis spectroscopy, performed in a UV-vis Specord 252 spectrophotometer (Analytik Jena, Jena, Germany), by monitoring the characteristic signal centered at 273 nm [23]. Spectroscopic data were converted into concentrations by using a calibration line, constructed according to our previous works [23,24,25]. Release tests were performed by immersing a pre-weighed sample (a disk having 25 mm diameter) in 10 mL of deionized distilled water (DDW) and then monitoring the absorbance of carvacrol at predetermined time intervals. After each measurement, the samples were immersed in fresh DDW to maximize the diffusion driving force. Thus, the cumulative release of carvacrol was calculated by adding (in sequence) the amount released after every step. It is worth noting that the experiments were conceived considering that the theoretical maximum releasable concentration (i.e., 0.3 mM) did not exceed the solubility of carvacrol in water (0.01 M).

Degradation tests were carried out on triplicates by immersing disk-shaped specimens (diameter 25 mm, thickness ~20 µm, weight about 8–10 mg), in a buffer solution at pH = 10 and 37 °C up to 864 h. At regular time intervals, the samples were washed with distilled water, followed by drying in vacuum oven for 24 h. Finally, the samples were weighed and the mass loss was determined as:

(1) $$M_{loss}=\frac{M_{dry,0}-M_{dry,t}}{M_{dry,0}}\times 100$$

## 3. Results and Discussion

**Figure 1:**
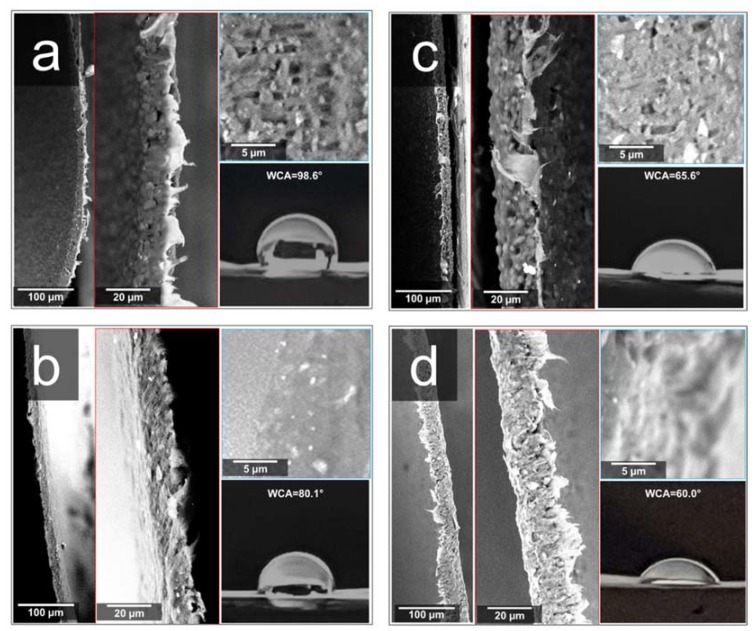
Cross-sectional SEM micrographs at different magnifications and water contact angles (WCAs) of (**a**) BF; (**b**) BF-D; (**c**) BF-C; (**d**) BF-D-C.

Figure 1 reports the cross-sectional SEM micrographs of films prepared at different magnifications, together with their WCA values. Neat BF (panel a) displays the typical morphology of polymer blends. Indeed, the islands and voids could be indicators of immiscibility and poor interfacial adhesion [10]. Submicrometric fillers can be recognized too. BF-D (panel b), exhibits a fairly well-dispersed nanoclay, since—at least at this scale—no evident aggregates were observed, and it was slightly more uniform in morphology. The presence of carvacrol (see Figure 1c,d), while leading to thicker films, did not significantly alter the morphology of the corresponding systems. It is, however, worth outlining that the sample BF-D-C displayed irregular edges because retained a remarkable ductility even after liquid nitrogen cooling. With respect to neat BF, possessing hydrophobic character (WCA = 98.6°), the materials containing D72T, carvacrol, or both additives displayed an increased hydrophilicity, with WCA values going from 80° (for BF-D) to 60° (for BF-D-C).

**Figure 2:**
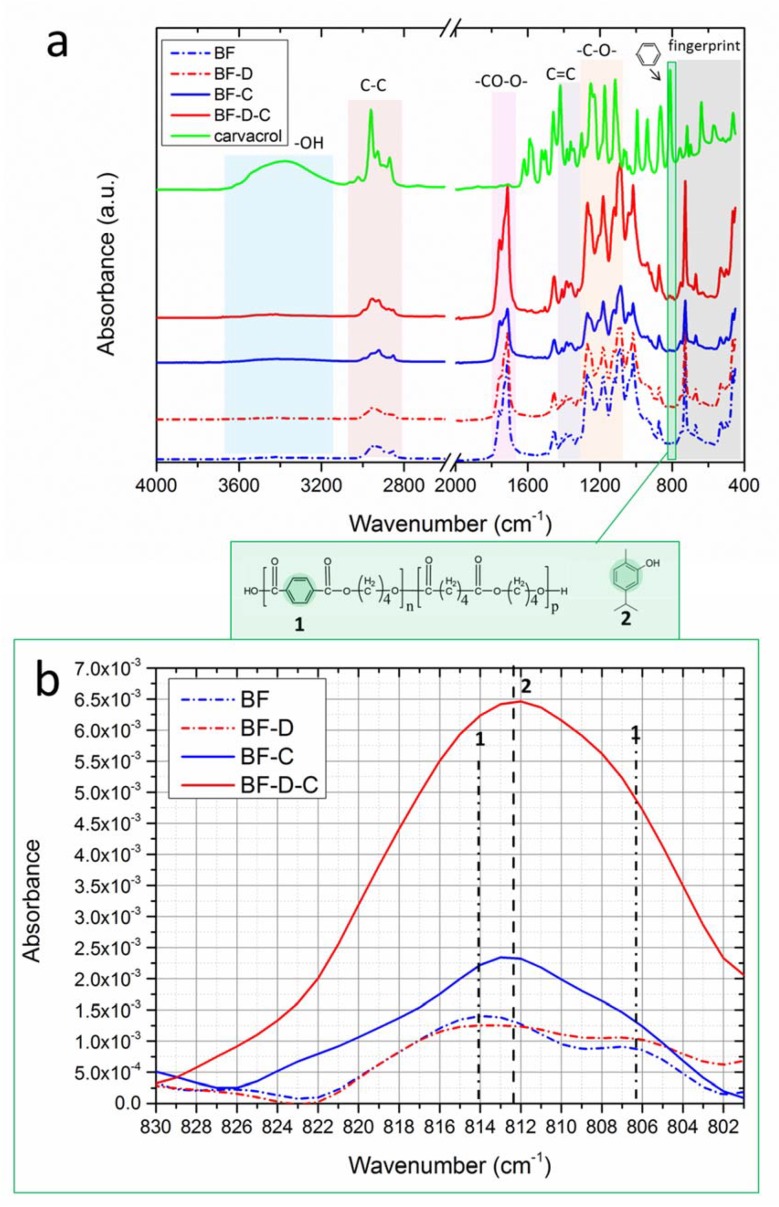
(**a**) FTIR/ATR spectra collected in the range 4000–450 cm^−1^; (**b**) close-up of the spectral range 830–800 cm^−1^ for the detection of carvacrol.

The results of FTIR/ATR spectroscopy are provided in Figure 2a,b. As discussed before, BF is a bioplastic whose formulation is proprietary. However, well-known absorption modes can be recognized, including the broad band at 3600–3000 cm^−1^ typically ascribed to OH-moieties, and the characteristic bands centered at 2957 and 2864 cm^−1^, assigned to C–H stretching of –CH_3_ groups. Moreover, a band at 1712 cm^−1^ can be attributed to C=O stretching, whereas the absorption band at 1455 cm^−1^ may refer to scissoring of –CH_2_ groups, and the modes located at 1412 cm^−1^ and 1378 cm^−1^ could be ascribed to C=C bonds typical of PBAT [26]. Furthermore, those centered at 1273, 1163, 1117, and 1104 cm^−1^ can be assigned to C-O stretching, while those located at 1016, 931, 867, and 729 cm^−1^ could refer to C-H bending [27,28,29,30]. 

The presence of carvacrol can be detected by monitoring the signal centered at ~812 cm^−1^ due to the characteristic aromatic ring of a polyphenol [23,24,25]. Aiming to evaluate the amount of carvacrol entrapped in the films, the spectral region 830–800 cm^−1^ of the spectra is provided in the panel b of the same figure [23,25]. Note that the spectra were normalized to the intensity of the peak centered at 753 cm^−1^, belonging to “fingerprint” region of PLA-phase of BF [23,24,25,31]. The results highlighted that carvacrol signal in BF-D-C has an intensity almost threefold that of BF-C. This feature may suggest that carvacrol amount is higher when D72T is used. This aspect is generally observed in nanocomposite systems, and it is attributed to the ability of some nanostructured fillers to block or at least mitigate the volatilization of carvacrol during processing, as investigated in our previous works [13,23,24,32]. However, due to the presence of the modes centered at 814 cm^−1^ and 806 cm^−1^ ascribed to the aromatic ring of PBAT, the bands were found to be overlapped. Hence, additional investigation on the actual amount of carvacrol in the samples will be provided in the following.

**Figure 3:**
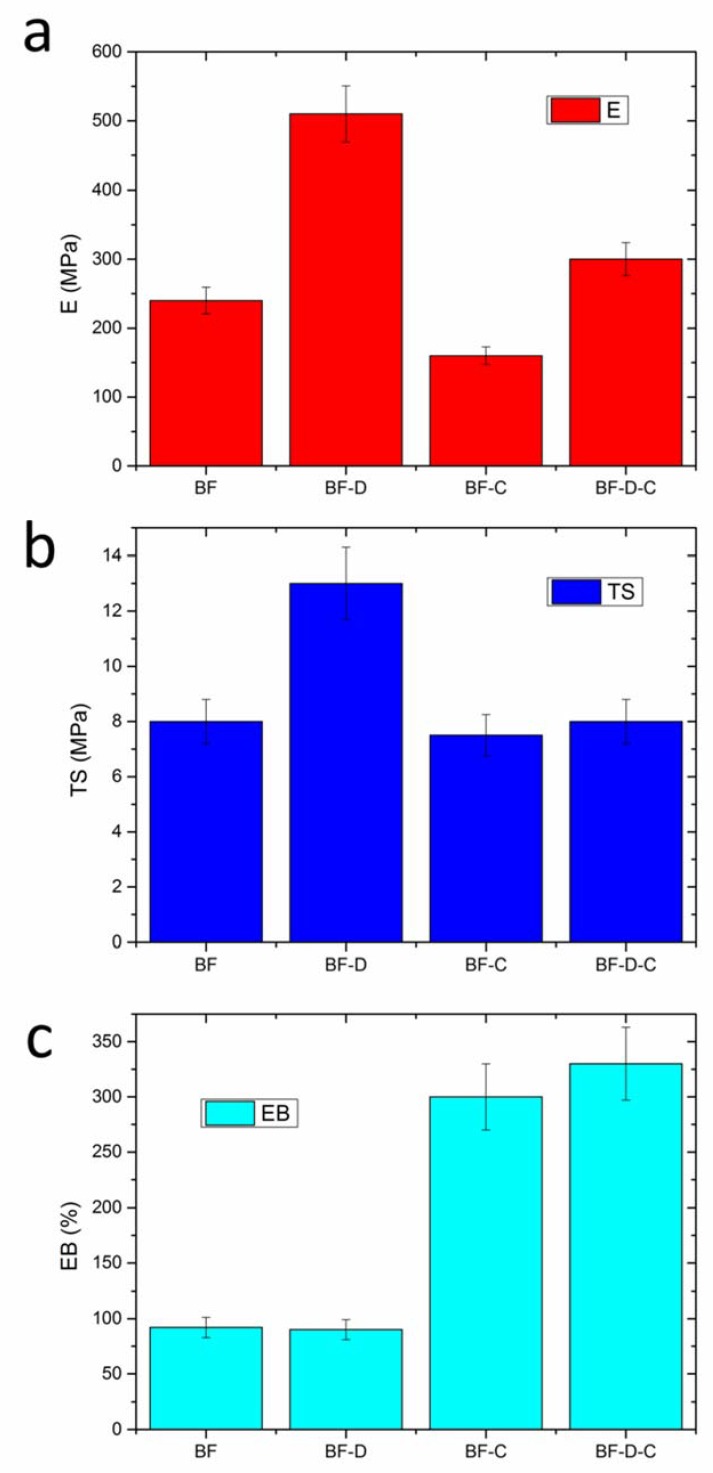
(**a**) Elastic modulus (E), (**b**) tensile strength (TS), and (**c**) elongation at break (EB) of each the four samples investigated.

The effects of nanoclay and carvacrol on the mechanical properties of BF films are shown in Figure 3a–c. The analysis of elastic modulus (E), Figure 3a, shows that the elastic modulus of BF is doubled in the presence of D72T, as is typically expected due to the incorporation of a rigid filler [33,34]. By contrast, adding carvacrol induced a drop in such property. In ternary systems (BF-D-C), the final effect depends on the interplay between stiffening effect of the clay and plasticizing effect exerted by carvacrol. Notably, BF-D-C displays an elastic modulus higher (+30% relative increment) than that of neat BF. TS (Figure 3b) proved to be practically unaltered for all the materials, except for BF-D, which displayed a +60% relative increment, likely owing to the strengthening effect of the clay.

As regards stretchability, Figure 3c, it is worth noting that the main effect on this property is induced by the presence of carvacrol, which led to huge increase of elongation at break with respect to the corresponding reference materials, likely due to its plasticizing effect. Furthermore, although deformability of BF-D proved to be slightly lower than that of BF, BF-D-C showed a ductility even higher than that of BF-C, likely owing to the higher carvacrol retention of BF-D-C films when compared to BF-C materials, as outlined in the discussion of FTIR/ATR results (see again Figure 2b).

**Figure 4:**
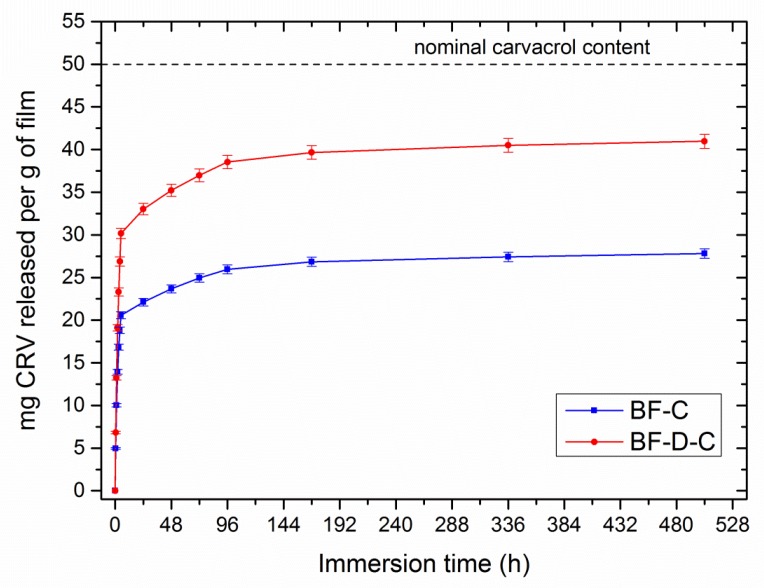
Amount of carvacrol released from BF-C and BF-D-C, expressed as milligrams of additive per one gram of film, plotted as a function of immersion time.

Figure 4 provides the amount of carvacrol released from BF-C and BF-D-C films. Both the materials showed a burst release at the early stages of the experiment, followed by a plateau, due to the progressive depletion of carvacrol, while differing from each other in the amount of additive released at saturation. In fact, BF-D-C released 40 mg of carvacrol per gram of film; i.e., almost two-fold that delivered by BF-C. That outcome further confirms the higher carvacrol retention of the systems containing nanoclay, in full agreement with the results of FTIR/ATR discussed above. It is worth noting that in both cases the final amount of carvacrol released at the equilibrium is lower than the nominal content in the films (i.e., 50 mg per 1 gram). This aspect, abundantly discussed in the scientific literature, can be ascribed to two phenomena [13,35,36,37]:

(i) Carvacrol tends to volatilize, especially at the high temperatures required for melt-processing;
(ii) A certain aliquot of carvacrol remains entrapped inside the matrix.

**Figure 5:**
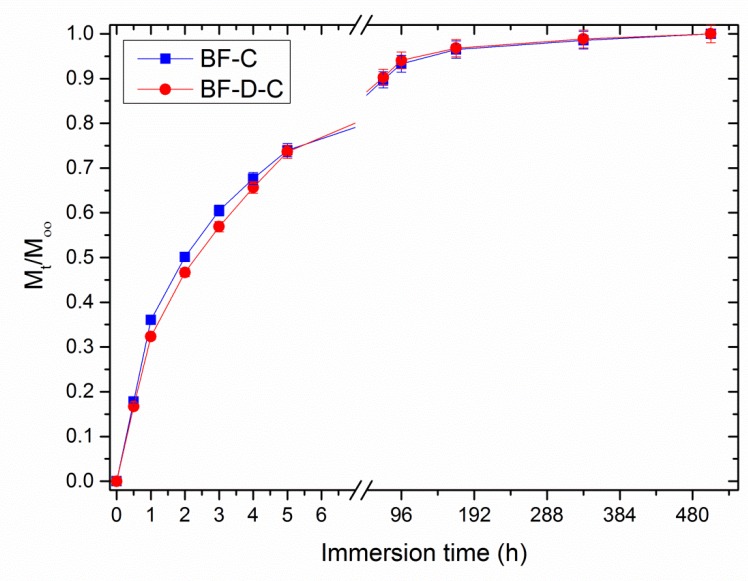
M_t_/M_∞_ as a function of immersion time for BF-C and BF-D-C samples.

Aiming to study the release kinetics of these materials, cumulative release data were normalized to the maximum value that each system achieved at saturation (i.e., at the end of the experiment). In fact, the aliquot of carvacrol released with respect to the maximum releasable amount can be expressed as the ratio between the carvacrol released at a given time (M_t_), and that released at equilibrium (M_∞_). Figure 5 reports the evolution of Mt/ M_∞_ as a function of immersion time in BF-C and BF-D-C systems.

Notably, the presence of nanoclay results in slightly slower release kinetics within the first 5 h, while negligible differences can be observed thereafter. This feature can be ascribed to the tortuosity imparted to carvacrol molecules by the presence of well-dispersed filler, which in turn forces the molecules to follow a longer path to leave the polymeric structure. For a closer inspection of this finding, experimental data plotted in Figure 5 were fitted according to Peppas-Korsmeyer model (Equation 2) [38]:

(2) $$\frac{M_{t}}{M_{\infty }}=kt^{n}$$

where k is a rate constant related to the properties of the drug delivery system and n is the diffusion exponent that is an indicator of the release mechanism. Indeed, when n ≤ 0.5, the drug release is Fickian, i.e., governed by diffusive phenomena, whereas it is dominated by swelling phenomena when n is equal to 1.0. Values of n between 0.5 and 1.0 indicate an anomalous transport, resulting from the combination of both phenomena. It is worth considering that this model is applicable only until M_t_/ M_∞_ = 0.6; i.e., before depletion. 

**Figure 6:**
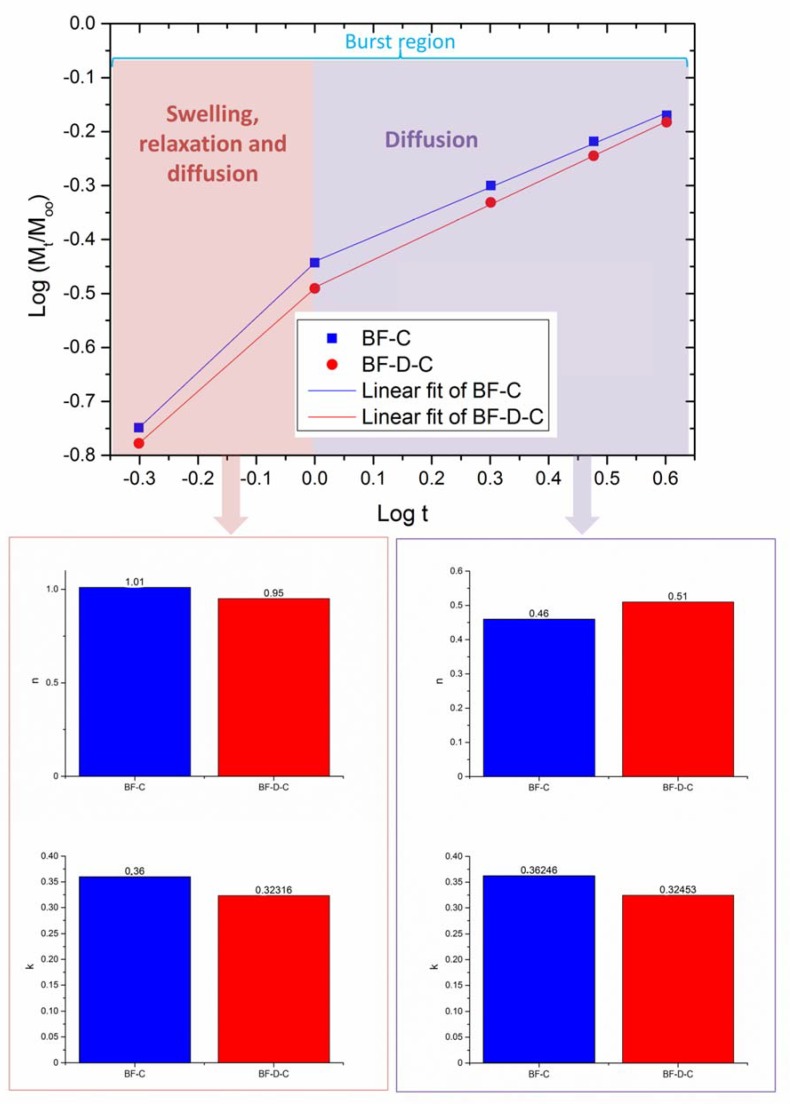
Log (M_t_/M_∞_) as a function of log time fitted according to Peppas-Korsmeyer model, together with *n* and *k* calculated in swelling and diffusive regions.

Plotting Log (M_t_/M_∞_) against log t, Figure 6, allows calculating n and k, respectively, as the slope and the intercept of a fitted line. It is worth outlining that linear fitting gave extremely satisfactory values of R^2^ (>0.99) by splitting such portions of the curves into two intervals, with the former one (until 1 h) being indicative of burst release, due to the partial swelling of the matrix and to the surface availability of carvacrol molecules readily releasable, and the latter one being governed by purely diffusive phenomena.

The analysis of n and k, whose values are reported in the subpanels of Figure 6, indicates that the release mechanisms (n) of the two systems are somehow similar, while being different in the kinetic constant, k, which proved to be lower in the case of BF-D-C samples, thereby suggesting that the geometric features of the samples being equal, the tortuosity imparted by the presence of the clay somehow slowed-down the additive release at the early stage of the test. 

Degradation under optimum conditions is another crucial prerequisite of green materials [39]. In order to assess the disintegrability of such systems under controlled conditions, hydrolytic degradation tests were performed at pH = 10 and mass loss percentage was plotted as a function of immersion time. It should be observed that degradation tests were performed at pH = 4 and pH = 7 also, showing only negligible mass losses, consistent with literature data. Therefore, they were omitted for the sake of brevity. 

**Figure 7:**
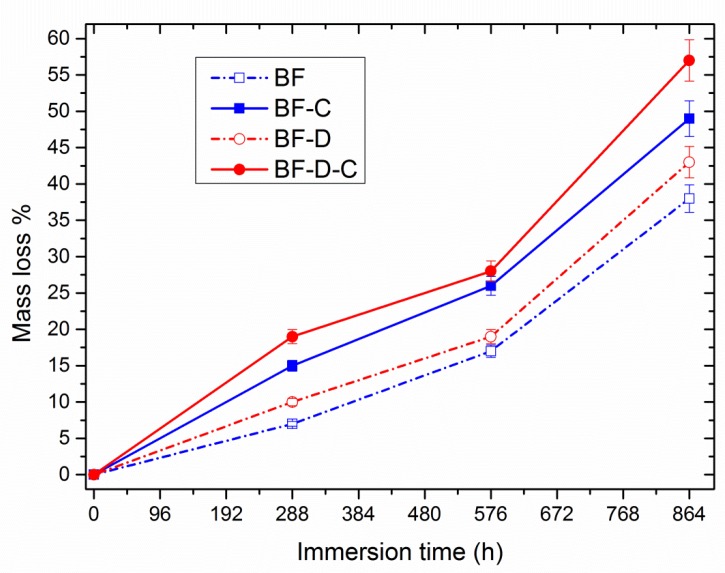
Mass loss percentage of the samples as a function of immersion time.

The results, provided in Figure 7, highlighted that all the samples exhibit a fairly fast degradation, with mass losses ranging from 37.5% to 57.5% depending on formulation. Notably, the presence of clay and/or carvacrol led to faster degradation rate, likely owing to a higher hydrophilic character, as confirmed by WCA tests [40]. Moreover, all the samples exhibited a speed-up in the degradation rate for time above 576 h, with this aspect—in biodegradable polyesters—usually being ascribed to the triggering of hydrolytic reactions in the bulk of the material after a certain induction time, with a consequent acceleration of disintegration rates [41]. It is of primary concern to point out that the hybrid loading of carvacrol and nanoclay resulted in the fastest degradation kinetics in the whole investigation.

The results obtained in this study remarked that ecofriendly materials endowed with tunable controlled release of antimicrobial additives can be easily achieved by film blowing, without using any solvent or time-consuming protocols. Furthermore, polymeric matrix, filler, and bioactive additive are naturally derived. The overall process is rapid and sustainable from both an economic and an environmental point of view. It is further worth noting that integrating a nanoclay results in a remarkable enhancement of mechanical properties, with stiffness relative improvements of up to +100%; i.e., comparable with the reinforcing effects typical of graphene derivatives, and higher than those of renewable nanofillers, including nanocellulose and lignin [20,23,42,43]. It is interesting to observe that nanoclay is crucial to avoid the most common issue related to systems containing carvacrol: i.e., volatilization of the latter during melt processing. In similar research, where other nanosized fillers or complexing agents were used to avoid carvacrol loss during melt processing, it was reported that the presence of graphene, owing to its extremely strong interaction with such molecules, proved to mitigate the burst release, while conversely diminishing the carvacrol amount delivered at saturation, with a consistent aliquot of polyphenols stacked onto graphenic planes via aromatic interactions [23]. By contrast, with respect to complexing agents, e.g., cyclodextrin, that require time-consuming preparation protocols, incorporating a nanoclay allows the direct melt-extrusion of polymer, additive, and filler [19]. 

The good stability of Bioflex^®^/nanoclay/carvacrol systems under acidic and neutral environments, and the evidence that the clay presence allows releasing higher amounts of carvacrol in a more controlled manner, makes them potentially suitable for food packaging, and for antimicrobial wrapping of various items [13]. 

The fast degradability of such systems in an alkaline environment (pH = 10) lets us envisage the possibility to disintegrate these materials in controlled conditions, whereas other nanofillers, such as nanocellulose, proved to delay the disintegration rates of biodegradable polyesters [44].

The remarkable release properties of such films could be useful for a strong antimicrobial activity, even relying on the synergy between antimicrobial nanoclays and carvacrol. Currently in-progress microbiological studies demonstrate that carvacrol exerts antimicrobial and antifungal activity even when released under the form of vapor, thereby potentially paving the road to the development of novel, contactless antimicrobial devices.

## 4. Conclusions

In this work, the possibility to prepare ecofriendly films based on a commercial PLA/PBAT blend, loaded with carvacrol, as an antimicrobial additive, and a nanoclay as a filler, was investigated. The presence of such hybrid loading did not alter the overall filmability of the polymer blend, while greatly enhancing the mechanical properties of such films, likely relying on the stiffening effect imparted by the nanoclay and the plasticizing effect of carvacrol. In fact, films containing both carvacrol and nanoclay, while retaining the same TS of neat matrix, displayed relative increments up to +70% and +200% in terms of elastic modulus and elongation at break, respectively. FTIR/ATR and release tests evidenced that the presence of nanoclay hindered carvacrol volatilization during processing, resulting in higher loading efficiency, and consequently, higher amounts of carvacrol released at the equilibrium. Furthermore, the tortuosity imparted by nanoclay mitigated the burst delivery of the antimicrobial additive, enabling a more controlled release. 

The disintegrability of such systems was evaluated by monitoring the mass loss as a function of immersion time at pH = 10. The results indicated that all the formulations displayed relatively fast degradation with mass loss values ranging from 37.5% to 57.5% after 876 h. However, the presence of clay and carvacrol accelerated the mass loss rate of Bio-Flex^®^, especially when added simultaneously. All the findings of this research show the tremendous potential of this hybrid loading in the development of green, sustainable, biodegradable films, particularly for suitable for food packaging applications, wrapping items, etc., thanks to the ecofriendly nature of starting materials. Of course, more focused studies on the actual antimicrobial properties of such systems in actual commercial applications should be performed.

## Figures and Tables

**Table 1:** Formulation, operating parameters used, and characteristics of the films prepared.

Sample	Carvacrol wt.% ^1^	D72T wt.% ^1^	Screw Speed (rpm)	T Profile (°C)	BUR ^2^	Thickness (μm)
BF	-	-	60	120-130-140-150-160	4	16 ± 3
BF-C	5	-	60	120-130-140-150-160	3.9	20 ± 2
BF-D	-	5	60	120-130-140-150-160	4.1	16 ± 2
BF-D-C	5	5	60	120-130-140-150-160	4	21 ± 2

^1^ Based on the total weight of the compounded materials. ^2^ Calculated as the ratio between the diameters of the bubble and of the die.
